# Participation experiences of people with deafblindness or dual sensory loss: A scoping review of global deafblind literature

**DOI:** 10.1371/journal.pone.0203772

**Published:** 2018-09-13

**Authors:** Atul Jaiswal, Heather Aldersey, Walter Wittich, Mansha Mirza, Marcia Finlayson

**Affiliations:** 1 School of Rehabilitation Therapy, Queen’s University, Kingston, Ontario, Canada; 2 School of Optometry, University of Montreal, Montreal, Quebec, Canada; 3 School of Physical and Occupational Therapy, McGill University, Montreal, Quebec, Canada; 4 Department of Occupational Therapy, University of Illinois at Chicago, Chicago, Illinois, United States of America; University of Birmingham, UNITED KINGDOM

## Abstract

**Background:**

Deafblindness, also known as dual sensory loss, is a varying combination of visual and hearing impairment in the same individual. Interest in this topic has increased recently due to evidence suggesting an increase in prevalence of this condition among older adults. Persons with deafblindness frequently experience participation barriers and social isolation. Developing an understanding of their experiences can inform the design of programs and policies to enhance participation of people with deafblindness in society.

**Objective:**

To identify and summarize available research literature on participation experiences of people with deafblindness or dual sensory loss.

**Methods:**

A comprehensive literature search of eight databases (CINAHL/EBSCO, Embase, ERIC, Global Health, MEDLINE, ProQuest, PsycINFO, PubMed) was performed in accordance with the Preferred Reporting Items for Systematic Reviews (PRISMA) during January 2017 and last updated in June 2017. In addition, non-peer reviewed (grey) literature was also retrieved in the form of online published reports of research projects by 16 deafblind-specific organizations across the globe. To be included, sources had to be published after 1990, had persons with deafblindness as the focal population, and focused on their participation experiences.

**Results:**

A total 1172 sources were identified of which 54 studies were included. The findings reveal that persons with deafblindness, regardless of origin of their impairment, experience difficulty in communication, mobility, daily living functioning, and social interactions. While these experiences may vary between individuals with congenital versus acquired conditions, they generally feel socially isolated, insecure and uncertain about their future.

**Conclusion:**

Participation experiences of persons with deafblindness are shaped by dynamic interactions between personal factors (such as onset and type of impairments) and environmental influences (such as attitude, technology, and supports). A better understanding of participation experiences may help professionals in placing emphasis on affected participation domains to design services to enhance participation of people with deafblindness.

## Introduction

Deafblindness, often a lifelong disability, is a combination of visual and hearing impairment in the same individual [1, 2]. Deafblindness is also known as dual sensory loss and ranges from mild loss in hearing and vision to total deafness and blindness depending upon its various combinations [2, 3]. Since our abilities to see and hear are complementary and enhance each other, this particular combination of hearing and vision dysfunction results in a unique condition that is more disabling than the sum of its impairments [3, 4]. The combined dysfunction “limits activities of a person and restricts full participation in society to such a degree that society is required to facilitate specific services, environmental alterations, and/or technology” [5]. Consequently, people with deafblindness frequently experience social isolation in their lives [2, 3, 6–8]

The deafblind population includes three distinct groups: Group 1 (people with congenital/pre-lingual deafblindness), Group 2 (people with acquired/post-lingual deafblindness–those who acquire both types of impairment during their lives or those with single sensory impairment [vision or hearing] by birth and then subsequently acquire another [vision or hearing] impairment), and Group 3 (dual sensory loss/impairment of vision and hearing due to age related changes in older adults) [3, 9, 10], each of which can vary in severity as well as order and time since onset.

The history of deafblindness can be traced back before the recognition of Helen Keller (1880–1968); yet, the development of research and practice specific to this population is still in its infancy [11–13]. The interest in this population has increased recently due to emerging evidence suggesting a rise in the prevalence of this condition among older adults worldwide [12, 14]. With the increase in older population globally, this rise in age-related deafblindness can be attributed to increased incidence of age-related sensorineural hearing loss, cataracts, glaucoma, and macular degeneration among older adults [3, 11, 12, 14].

Participation is a highly valued goal in rehabilitation for persons with disabilities, including those with deafblindness [15–17]. Furthermore, international legislations such as the United Nations Convention on the Rights of Persons with Disabilities (UNCRPD) recognize the right of all persons with disabilities to fully participate in society and acknowledge the distinct needs of persons with deafblindness [18]. Therefore, it is vital to explore the participation experiences of people with deafblindness. A better understanding of deafblind individuals’ experiences can help rehabilitation professionals and policy makers design programs and policies to enhance societal participation of persons with deafblindness.

## Methods

This scoping review was based on the methodological framework published by Arksey and O’Malley [19] with the addition of recommendations from scoping review methodology scholars including Levac and colleagues [20] and Peters and colleagues [21]. Scoping review is defined as a form of knowledge synthesis on a particular research area to identify key concepts, types of evidence, and gaps in the research literature by systematically searching and synthesizing existing knowledge to inform health care practice, policy, and research [22, 23]. The purpose of this review was to map the literature on participation experiences of persons with deafblindness and summarize a range of evidence that could inform the programs and policies for rehabilitation of persons with deafblindness.

The Arksey & O’Malley framework includes five stages: identifying the research question, identifying relevant studies, selecting studies, charting the data, and then collating, summarizing, and reporting the results. We enhanced rigor in our methods by creating a linkage between the purpose and research question; ensuring breadth and comprehensiveness of the scoping process; using an iterative approach in study selection and extraction; including a descriptive numerical summary and qualitative thematic analysis while reporting results, and discussing the implications for future policy, practice, or research. [20]. We also incorporated recommendations of Peters and colleagues by reporting operational definition of ‘population’, ‘concept’, and ‘context’ of the review; search strategy; research database searched; details of the criteria used for inclusion and exclusion of studies; and how the data will be extracted and mapped [21].

### Stage 1: Identifying the research question

The review was guided by the research question, ‘What does the existing literature report about the experiences of participation for persons with deafblindness in society?’ For the purpose of this study, the population ‘persons with deafblindness’ are individuals with a varying combination of visual and hearing impairment [3, 11]. The concept of ‘participation’ was defined as involvement in any life situations [24]. The spatial context for this study was societies around the world where persons with deafblindness live, whereas the temporal context was post 1990; a unanimous resolution was passed in the 1990 Conference of the International Association for the Education of Deafblind People (currently known as Deafblind International) to create the unified term “deafblind” recognizing the unique nature of this disability [2, 25, 26].

### Stage two: Identifying relevant studies

A database search strategy was developed by the research team in consultation with a senior health science librarian from Queen’s University. A comprehensive literature search of eight databases (CINAHL/EBSCO, Embase, ERIC, Global Health, MEDLINE, ProQuest, PsycInfo, and PubMed) was performed in January 2017 and last updated in June 2017 in accordance with the Preferred Reporting Items for Systematic Reviews (PRISMA) [27] (see Table 1).

**Table 1:** Sources and search terms.

Research databases searched	Search terms
• Cumulative Index to Nursing and Allied Health Literature (CINAHL) • Education Resources Information Centre (ERIC) • Embase • Global Health • MEDLINE • ProQuest • PsycINFO • PubMed	• (deafblind* OR deaf-blind* OR (“deaf and blind") OR "dual sensory loss" OR "dual sensory impairment" OR("combined hearing and visual impairment") OR ("combined hearing and visual loss") AND (experience* OR engage* OR participat* OR involve*) AND (society OR community)
Other sources including • Google and Google Scholar • Journal of Deaf Studies & Deaf Education • Journal of Visual Impairment & Blindness • Online content of development organizations	• deafblind AND experiences • deafblind AND experiences • deafblind AND experiences • deafblind AND experiences

In addition, non-peer reviewed (grey) literature was also retrieved in the form of online published reports of research projects by 16 deafblind-specific organizations around the world (see Table 2). Grey literature was included to maximize the comprehensiveness of the study following the recommendations of Peters and colleagues [21]. The selection of these organizations was based on the availability of research reports on their website. Reports were accessed directly from the organizations’ websites. Search terms were based on the key concepts drawn from the review question and its context (see Table 1). The log book used for the review is available upon request.

**Table 2:** List of organizations searched for grey literature.

SN	Organization	Website
1	European Union	https://europa.eu/
2	National Centre on Deaf-blindness	https://nationaldb.org/
3	Sense UK	https://www.sense.org.uk/
4	Deafblind UK	http://deafblind.org.uk/
5	Senses Australia	https://www.senses.org.au/
6	Sense International	https://www.senseinternational.org.uk/
7	Deafblind International	http://www.deafblindinternational.org/
8	World Federation of the Deafblind	http://www.wfdb.eu/
9	Helen Keller National Center for Deaf-Blind Youths & Adults	https://www.helenkeller.org/
10	Perkins School for the Blind	http://www.perkins.org/
11	American Association of the Deaf-Blind	http://www.aadb.org/
12	Canadian Deafblind National Association	http://www.cdbanational.com/
13	Deafblind Ontario	http://www.deafblindontario.com/
14	Helen Keller Institute for Deaf & Deafblind, India	http://helenkellerinstitutefordeafanddeafblind.org
15	Perkins Voice and Vision India	http://www.voicevisionindia.org/
16	Sense International India	http://www.senseintindia.org/

The research team conducted a web search in Google and Google Scholar using keywords ‘deafblind’ and ‘experiences’ to identify any literature that had not been captured in original search. An *a priori* decision was made to screen only the first 100 hits (most relevant). In addition, reference lists of the most relevant articles were manually reviewed to identify additional studies, a technique called *snowballing*. Later, the team manually searched the online databases of Journal of Visual impairment and Blindness (JVIB) and Journal of Deaf studies and Deaf education (JDSDE) published since 1990. These journals were chosen due to their relevance to the field, the likelihood of finding a greater number of relevant articles, and the fact that not all their content is indexed electronically. The researchers retrieved and imported all relevant sources into the bibliographic manager Mendeley Desktop (2016) (Version 1.17.6.), and removed duplicates.

### Stage three: Study selection

A two-stage screening process was used to assess the relevance of studies–first, at the level of title/abstract, and second, at the level of full-text review. Consistent with the scoping review process, *post hoc* inclusion/exclusion criteria (see Table 3) were established to assess the relevance of studies identified in the search. Associated disability conditions (such as autism, cerebral palsy, cognitive impairment, etc.) were not included because of the likelihood that these co-existing conditions might influence the participation experiences of individuals with deafblindness. It is important to note that studies were not excluded if they had focus on experiences of persons with deafblindness/dual sensory loss, but might have also included non-disabled people or persons with single sensory impairment in their study sample along with individuals with deafblindness/dual sensory loss.

**Table 3:** Inclusion and exclusion criteria.

Inclusion Criteria	Exclusion Criteria
• Studies related to persons with deafblindness or dual sensory loss and their experiences of involvement in any life situations. • Study population comprised with deafblindness irrespective of their age. • Sources that were qualitative and quantitative studies, literature reviews, research reports, and personal accounts. • Studies that were produced from 1990 to date.	• Studies related to persons with deafblindness with co-existing conditions such as autism, cerebral palsy, cognitive impairment, etc. • Study population comprised parents, or professionals. • Study that focused solely on medical/rehabilitation interventions or educational programs. • Full text of the study was not available in English. • Study that lacked any clarity in relation to experiences of persons with deafblindness

**Fig 1:**
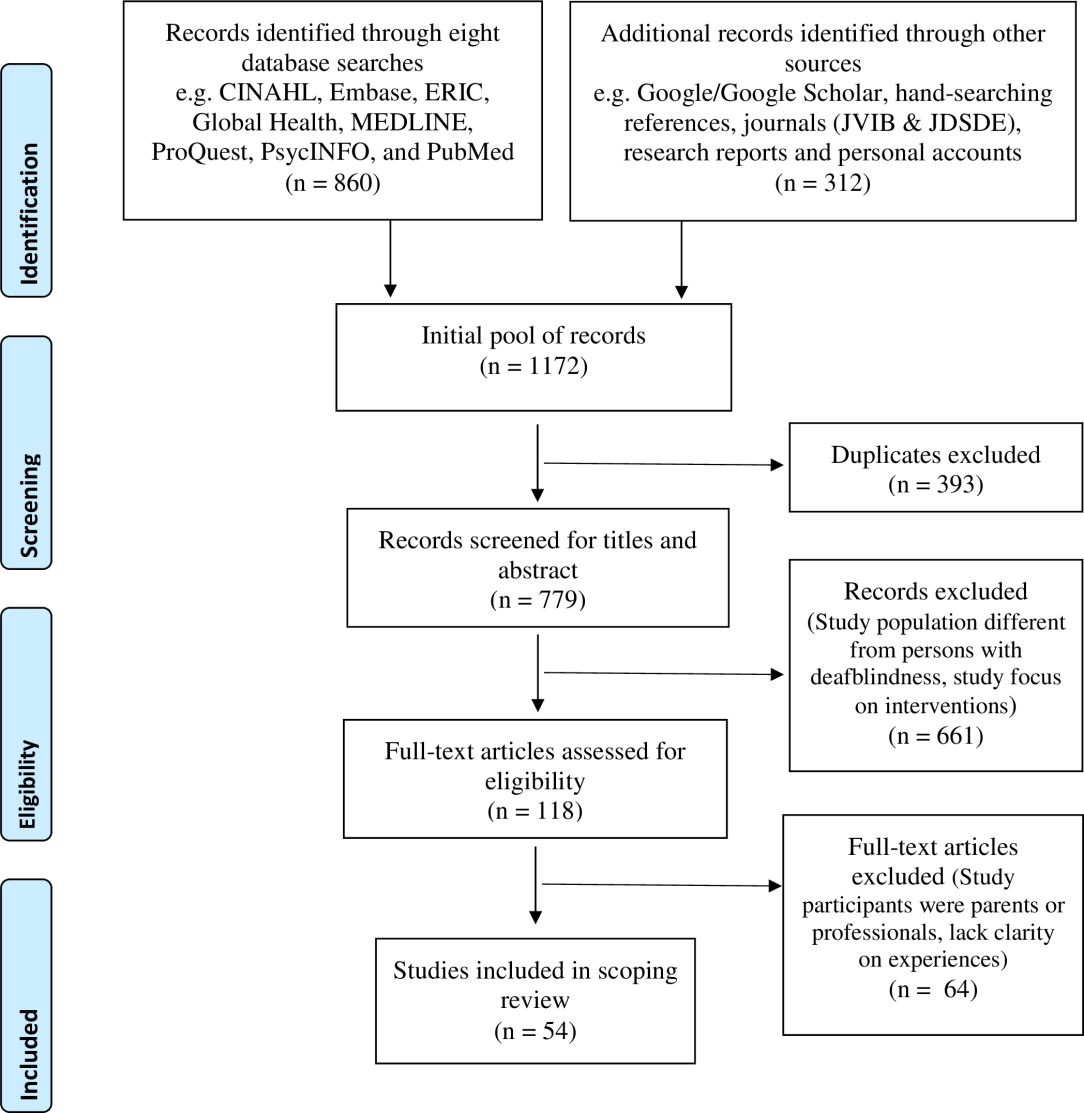
PRISMA flow diagram.

Eligible sources were reviewed in full and a final list of sources was compiled in a spreadsheet using Microsoft Excel 2013. Two authors (A.J. and H.A) reviewed the compiled list of articles/sources and the other authors (M.M., W.W., and M.F.) were consulted for disagreements until consensus was reached. Details on identification, screening, eligibility and inclusion can be found in the PRISMA flow diagram (Fig 1) [27]. PRISMA 2009 checklist can be found in S1 File.

### Stage four: Charting the data

The first and second authors held several meetings to discuss the list of descriptors in the data charting form in the spreadsheet. Extracted data included details regarding name of authors, year of study, journal title, location of study, study population, number of participants, focus of article, aim of the study, methodology used, key outcome, and any other data significant to the scoping question. The same format was used for grey literature, and ‘not available’ was used if the information was missing from the source.

### Stage five: Collating, summarizing, and reporting the results

Consistent with recommendations of Arksey and O’Malley’s, and Levac and colleagues, results were reported using descriptive numerical summary and qualitative thematic analysis [19, 20]. A summary of descriptive findings was collated from the spreadsheet and presented in the form of tables and figures. Braun and Clarke’s [28] principles of thematic analysis were used to identify key themes from the extracted data by the first author (A.J.) and reviewed by the other authors to confirm the interpretation. Authors discussed findings to develop an overall understanding of deafblind research worldwide, and its major focus, key challenges, limitations, and gaps with reference to the experiences of persons with deafblindness.

## Results

Out of 1172 sources identified, 860 sources were extracted from eight bibliographic databases; 286 were included from Google/Google Scholar, hand-searching references, and two academic journals (JVIB and JDSDE); and 26 research reports from deafblind-specific organizations. Fifty-four sources were ultimately accepted that met all selection criteria (see Table 3). Information on number of sources identified, screened, found eligible and finally included in the study can be found in Fig 1 - PRISMA flowchart.

### Characteristics of the records included in the study

Of 54 sources that were included in the final review, the majority were empirical studies (n = 36), followed by reviews (n = 12), research reports (n = 4), and personal narratives (n = 2). The records presenting empirical research covered a broad spectrum of methodologies (e.g. quantitative, qualitative and mixed), designs (e.g. longitudinal, cross-sectional, case study, and participatory action research) and data collection strategies (e.g. interviews, focus groups, observations, short films/videos, document reviews, and surveys). The non-empirical records were primarily literature reviews, research reports, and personal narratives. Table 4 provides details on study location, publication year and name of publication journal, while Table 5 provides an overview of selected studies with the study aim, focus, participant population and sample size. It is worthwhile to mention that the study population described in Table 5 includes different terminologies such as deafblindness, dual sensory impairments, and dual sensory loss that were reported in the respective studies.

**Table 4:** Characteristics of records included in the study (n = 54).

Authors	Year of publication	Journal details	Location of Study
Berry, P. et al.,	2004	Care Management Journals	United States
Bodsworth, S.M. et al.	2011	British Journal of Visual Impairment	United Kingdom
Brennan, M., & Bally, S.J.	2007	Trends in Amplification	United States
Brennan, M., Horowitz, A., & Su, Y.P.	2005	Gerontologist	United States
Brennan, M.	2003	Generations	Not specified
Bruce, S.M., & Parker, A.T.	2012	American Annals of the Deaf	United States
Bruce, S.M., Zatta, M.C., & Gavin, M.	2016	Journal of Visual Impairment & Blindness	United States
Capella-McDonnall, M.E.	2005	International Journal of Geriatric Psychiatry	United States
Cimarolli, V.R., & Jopp, D.S.	2014	Age and Ageing	United States
Correa-Torres, S.M.	2008	Journal of Visual Impairment & Blindness	United States
Crews, J.E., & Campbell, V.A.	2004	American Journal of Public Health	United States
Dalby, D.M. et al.,	2009	Journal of Visual Impairment & Blindness	Canada
Damen, G.W. et al.,	2005	International Journal of Rehabilitation Research	Europe (France, Germany, Ireland, Italy, Spain, UK, and The Netherlands)
Dammeyer, J.	2012	International Journal of Pediatric Otorhinolaryngology	Denmark (Europe)
Dammeyer, J.	2014	Scandinavian Journal of Public Health	Not specified
Danermark, B.D., & Moller, K.	2008	International Journal of Audiology	Not specified
Deafblind UK	2007	Research Report	United Kingdom
Ellis, L., & Hodges, L.	2013	Research report	United Kingdom
Emerson, J., & Bishop, J.	2012	Journal of Visual Impairment & Blindness	United States
Figueiredo, M. Z. de A. et al.,	2013	CoDAS	Brazil
Fletcher, P.C., & Guthrie, D.M.	2013	International Journal of Disability, Community & Rehabilitation	Canada
Fukushima, S.	2011	Personal Accounts in an Edited book	United Kingdom
Gibson, J.	2000	Journal of Adventure Education & Outdoor Learning	United Kingdom
Gopinath, B. et al.,.	2016	Age and Ageing	Australia
Gribs, H., & Others.	1995	Journal of Visual Impairment & Blindness	United States
Guthrie, D.M. et al.,	2016	PLOS One	Europe & Other two countries (Finland, Belgium, Canada, and US)
Heine C., & Browning, C.J.	2002	Disability and Rehabilitation	Not specified
Heine, C., & Browning, C.J.	2015	Gerontologist	Not specified
Heine, C., & Browning, C.J.	2014	Frontiers in Aging Neuroscience	Not specified
Heine, C., & Browning, C.J.	2004	Ageing and Society	Australia
Hersh, M.A.	2013	Journal of Deaf Studies and Deaf Education	Europe (Czech, France, Italy, Poland, UK, and Spain)
Hersh, M.A.	2013	Technology and Disability	Europe (Czech, France, Italy, Poland, UK, and Spain)
Högner, N.	2015	Journal of Visual Impairment & Blindness	Germany (Europe)
Ingraham, C.L., & Others.	1995	Journal of Visual Impairment & Blindness	United States
Kamenopoulou, L.	2012	British Journal of Special Education	United Kingdom
Lieberman, L., & Stuart, M.	2002	Journal of Visual Impairment & Blindness	Not specified
Lieberman, L.J., Haegele, J.A., & Marquez, M.	n.d.	Collection of case stories	United States
Lieberman, L.J., & MacVicar, J.M.	2003	Journal of Visual Impairment & Blindness	United States
McDonnall, M.C. et al.,	2016	Journal of Visual Impairment & Blindness	United States
Miner, I.D.	1995	Journal of Visual Impairment & Blindness	United States
Möller, K., & Danermark, B.	2007	American Annals of the Deaf	Sweden (Europe)
Reid, C.	2010	Journal of Media and Culture	United States
Rönnberg, J., & Borg, E.	2001	Scandinavian Audiology	Not specified
Saunders, G.H., & Echt, K.V.	2007	Trends in Amplification	Not specified
Schneider, J.M. et al.,	2011	Journal of Aging and Health	Not specified
Sense UK	n.d.	Research report	United Kingdom
Simcock, P.	2016	Health & Social Care in the Community	Not specified
Simcock, P.	2016	Ageing and Society	United Kingdom
Simcock, P., & Manthorpe, J.	2014	British Journal of Social Work	United Kingdom
Soper, J.	2006	British Journal of Visual Impairment	United Kingdom
Viljanen, A. et al.,	2014	European Journal of Ageing	Europe (11 countries)
Wahlqvist, M., Moller, C., & Moller, K.	2013	Journal of Visual Impairment & Blindness	Sweden (Europe)
Watters, C., Owen, M., & Munroe, S.	2005	Research report	Canada
Yamada, Y. et al.,.	2016	The Journals of Gerontology	Europe and Israel (the Czech Republic, England, Finland, France, Germany, Israel, Italy, and the Netherlands)

**Table 5:** Overview of records included in the study (n = 54).

References	Study population	Type of disability	Number of participants	Focus of article	Aim of the study
Kamenopoulou, L. (2012)	Students with Db^a^	Not specified	4	Inclusion of students with deafblindness in mainstream schools	To explore the social inclusion and participation of four Db pupils in mainstream placements
Dalby et al., (2009)	Adults with Db	Mixed (Grp^a^ 1 and Grp 2)	182 (Congenital-88, and 94 acquired) (94 M and 88 F)	Characteristics of individuals with congenital and acquired deaf-blindness	To compare participants with congenital and acquired Db
Gribs et al., (1995)	Older woman with Db	Acquired (Grp 2)	1	Life story of 87 year old lady	To understand her life in school, work, and other life domains.
Wahlqvist et al., (2013)	Adults with Db	Usher Syndrome (Ush^a^ Type II (Acquired) (Grp 2)	96	Physical and psychological health	To describe the physical and psychological health of persons with Usher syndrome Type II (Ush2) and to explore any differences in terms of gender.
Lieberman, L.J., & MacVicar, J.M. (2003)	Young adults with Db	Not specified	54 (34M and 20F)	Play and Recreation	To analyze the current recreational practices and barriers faced by 54 youths with Db
Miner, I.D. (1995)	Adults with Db	Usher Syndrome Type I (Acquired) (Grp 2)	39 (20F and 19M)	Psychological implications of Usher syndrome	To investigate psychological implications of Usher syndrome throughout the lifecycle
Bruce et al., (2016)	Students with or without Db	Congenital (Grp 1)	6	Socialization skills	To study socialization skills in six children with Db in the context of an arranged interaction space
Correa-Torres, S.M. (2008)	Students with Db	Not specified	3 students, their mothers, teachers, and intervenors	Social experiences and opportunities for communication	To investigate the nature of social experiences and opportunities for communication among students with Db, their sighted peers, and adults in inclusive settings.
Ingraham et al., (1995)	Students with Db	Not specified	3 students	Social interactions	To explore the social interactions of three gifted students with Db
Emerson, J., & Bishop, J. (2012)	Students with Db	Not specified	10 students	Access and communication using technology	To investigate the potential for increasing access and communication using videophone technology
Bruce, S.M., & Parker, A.T. (2012)	Young adults with Db	Mixed (Grp 1 and Grp 2)	6	Experience of process of change	To learn more about how the youth with Db experienced the process of becoming change agents in the advocacy course
Yamada et al., (2016)	Nursing home residents with or without DSI^a^	Acquired (Grp 3)	Total—1989; DSI-122	Cognitive decline and social engagement	To examine whether nursing home residents with DSI have a greater cognitive decline over time and whether social engagement modifies this association.
Gopinath et al., (2016)	Older Adults with or without DSI	Acquired (Grp 3)	Total 1478 older adults (DSI not specified)	Dual sensory impairment (DSI) and incidence of falls	To assess the association between dual sensory impairment (DSI) and incidence of falls
Cimarolli, V.R. & Jopp, D.S. (2014)	Oldest Old Adults with or without DSI	Acquired (Grp 3)	119	Prevalence of SI and association with functional disability	To explore associations of sensory impairments with functional disability in near-centenarians and centenarians
Dammeyer, J. (2012)	Children with Db	Mixed (Grp 1 and Grp 2)	26 (Ush) and 17 (CHARGE)	Developmental characteristics of children with Usher syndrome and CHARGE syndrome	To describe the developmental characteristics of children with Usher syndrome and CHARGE syndrome
Schneider et al., (2011)	Older adults with DSI	Acquired (Grp 3)	Not applicable	Dual sensory impairment in older age	To examine the frequency and effects of DSI in older age
Heine C. & Browning C.J. (2002)	Older adults with sensory loss	Acquired (Grp 3)	Not applicable	Sensory loss in older adults	To understand the communication and psychosocial consequences of sensory loss in older adults
Heine, C., & Browning C.J. (2015)	Older adults with DSL	Acquired (Grp 3)	Not applicable	Effect of dual sensory loss in older adults	To critically evaluate the evidence from studies that examined dual sensory loss and its effects on older adults.
Dammeyer, J. (2014)	Not specified	Not specified	Not applicable	Literature review on deafblindness	To review literature on Db
Danermark, B.D., & Moller K. (2008)	NA	Not specified	NA	Information and communication	To give an introduction to Db in relation to trust, ontological security, social recognition and self-identity
Brennan, M., & Bally, S.J. (2007)	Older adults with DSL	Acquired (Grp 3)	Not applicable	Psychosocial Adaptations to DSI	To review the prevalence and causes of dual impairment and its effects on functioning for both individuals affected and their families
Saunders, G.H., & Echt, K.V. (2007)	Older adults with DSI	Acquired (Grp 3)	Not applicable	Effect of DSI	To present overview of DSI and research needs regarding rehabilitation strategies
Damen, G.W. et al., (2005)	Adults with Ush	Usher Syndrome Type I, II, and III (Acquired) (Grp 2)	93 (50M & 43F) (Ush)	Maintaining independence—access to information, communication and mobility	To understand the challenges faced by Usher patients in order to maintain independence with progressing age and increasing hearing/RP^a^ difficulties
Brennan, M., Horowitz, A., & Su, Y.P. (2005)	Older Adults with or without DSL^a^	Acquired (Grp 3)	5151	DSL and functional competence	To examine the relation of dual sensory loss to functional competence among older adults
Simcock, P. (2016)	Persons with Db	Not specified	Not applicable	Vulnerability of persons with Db	To synthesize existing knowledge about the relation-ship between Db and vulnerability
Capella-McDonnall, M.E. (2005)	Older adults with or without DSL	Acquired (Grp 3)	9832	DSL and depression	To determine the effect of dual sensory loss (i.e. combined hearing and vision loss) on depressive symptoms
Hersh, M.A. (2013)	Adults with Db	Mixed (Grp 1 and Grp 2)	27 Db +1 mother of autistic Db	Experiences of communication, independence, and isolation	To discuss issues related to communication, independence, and isolation
Berry et al., (2004)	Older adults with DSL	Acquired (Grp 3)	Not applicable	Challenges due to DSI	To outline the main issues faced by older individuals who experience dual sensory impairments
McDonnall et al., (2016)	Older Adults with or without DSI	Acquired (Grp 3)	131	Needs and challenges of seniors with dual sensory loss	To identify the needs and challenges of seniors with dual sensory loss
Viljanen, A. (2014)	Older adults with or without DSI	Acquired (Grp 3)	27536	Prevalence and association with social inactivity	To describe the prevalence of sensory difficulties and whether sensory difficulties are associated with social inactivity in older Europeans
Brennan, M. (2003)	Older Adults with DSL	Not specified	Not specified	Prevalence and impact on quality of life	To describe prevalence and impact on quality of life due to DSL
Högner, N. (2015)	Adults with Ush	Usher Syndrome Type II (Acquired) (Grp 2)	262 (139F and 123M)	Psychological Stress	To assess stress in people with Usher Syndrome type II and the influence of personal variables such as age, gender, and employment on stress
Guthrie et al., (2016)	Older adults with or without DSI	Acquired (Grp 3)	Not specified	Demographic characteristics, functional and psychosocial outcomes	To compare older adults with DSI to all others across demographic characteristics, functional and psychosocial outcomes.
Heine, C., & Browning, C. J. (2004)	Older Adults with DSL	Acquired (Grp 3)	10 (8F and 2M)	Communication and psychosocial perceptions	To explore the communication and psychosocial perceptions of a group of older adults with single or DSL
Lieberman, L., & Stuart, M. (2002)	Adults with Db	Mixed (Grp 1 and Grp 2)	51 (25M and 26F)	Leisure and recreation	To describe leisure and recreation for persons with Db
Rönnberg, J., & Borg, E. (2001)	Persons with Db	Mixed (Grp 1 and Grp 2)	Not applicable	Behavioral and communicative research on deaf-blind individuals	To present primarily the last 10–20 years of behavioral and communicative research on Db individuals
Möller, K., & Danermark, B. (2007)	Students with Db	Acquired (Grp 2)	34	Personal and environmental factors affecting participation	To describe environmental and personal factors that, from the student perspective, impede participation in education in secondary upper schools by students with post lingual Db
Soper, J. (2006)	Adults with Db	Acquired (Grp 2)	5	Experience of cochlear implantation	To examine the experience of cochlear implantation in individuals with acquired Db, focusing on access to information, communication, and mobility.
Simcock, P., & Manthorpe, J. (2014)	Young Woman with Db	Congenital (Grp 1)	1	Experience of vulnerability	To explore this unique impairment in the illustrative case of Beverley Lewis
Bodsworth et al., (2011)	Adults with DSI	Not specified	539	Mental health experiences	To document psychological distress and unmet need among adults with DSI
Deafblind UK. (2007)	Adults with Db	Not specified	486	Experiences of access to health services	To understand experiences of access to health services
Ellis, L., & Hodges, L. (2013)	Adults with Ush 1, 2, and 3	Acquired (Grp 2)	42 (16M and 26F)	Experiences of diagnosis	To explore experiences of being diagnosed with Usher and impact of diagnosis on the lives and experiences of people with Usher syndrome
Watters, C., Owen, M., & Munroe, S. (2005)	Adults with Db, their parents, and professionals	Mixed (Grp 1 and Grp 2)	Total -3306; 44 (Adults with Db)- 42 acquired + 2 congenital; (29F & 15M)	Demographics, needs, and issues	(1)To provide demographic information about persons with congenital and acquired Db in Canada; (2) To discuss service needs of persons with Db and their parents; (3) To present an overview of the personal stories of the barriers and successes experienced by them; (4) To outline existing services; and (5) To provide directions for future research.
Figueiredo et al., (2013)	Adults with Ush	Acquired (Grp 2)	11	Impacts of the disease on their daily lives	To characterize communication and main mechanisms that facilitate interpersonal relationships of Db, especially in relation to communication and locomotion and the impact of these aspects on Db.
Fletcher, P.C., & Guthrie, D.M. (2013)	Adults with DSL	Acquired (Grp 2)	7	Lived experiences of challenges associated with acquired Db	To examine the lived experiences of people with acquired Db
Crews, J.E., & Campbell, V.A. (2004)	Older adults with or without DSI	Acquired (Grp 3)	Total 9447; 779 (Vision and hearing loss)	health, activity, and social participation	To investigate the health, activity, and social participation of people aged 70 years or older with vision impairment, hearing loss, or both.
Heine, C., & Browning, C.J. (2014)	Older adults with DSL	Acquired (Grp 3)	Not applicable	mental health experiences	To examine the mental health of older adults with DSL
Simcock, P. (2016)	Older adults with Db	Acquired (Grp 3)	Not applicable	experience of ageing	To explore the experiences of those ageing with Db
Hersh, M.A. (2013)	Adults with Db	Mixed (Grp 1 and Grp 2)	27 Db +1 mother of autistic Db	Experiences of stigma in using assistive devices for communication and mobility	To discuss issues, including stigma, related to the use of assistive communication and mobility devices
Fukushima, S. (2011)	Adult with Db	Acquired (Grp 2)	1	Experience of deafblindness and disability studies	To characterize disability studies based on the author’s own experience as a Db person
Gibson, J. (2000)	Adult with Db	Congenital (Grp 1)	1	Experiences of Outdoor Activities	To document the experiences of a deafblind adult in a variety of outdoor activities in holidays.
Lieberman et al., (n.d.)	Adults with Db	Mixed (mostly Ushers—Grp 2	18	Experience of recreation	To highlight recreational and leisure pursuits for individuals who are deafblind
Reid, C. (2010)	Adult woman with Db	Acquired (Grp 2)	1	Personal experience as a deafblind	To narrate the life journey
Sense UK. (n.d.)	Adults with Db	Not specified	8	Experience of transition into adulthood and associated	To understand the experiences of transition process for young deafblind people.

^a^Db means deafblindness; Ush means Usher syndrome; DSI means dual sensory impairments; DSL means dual sensory loss; RP means Retinitis Pigmentosa and Grp means group

**Fig 2:**
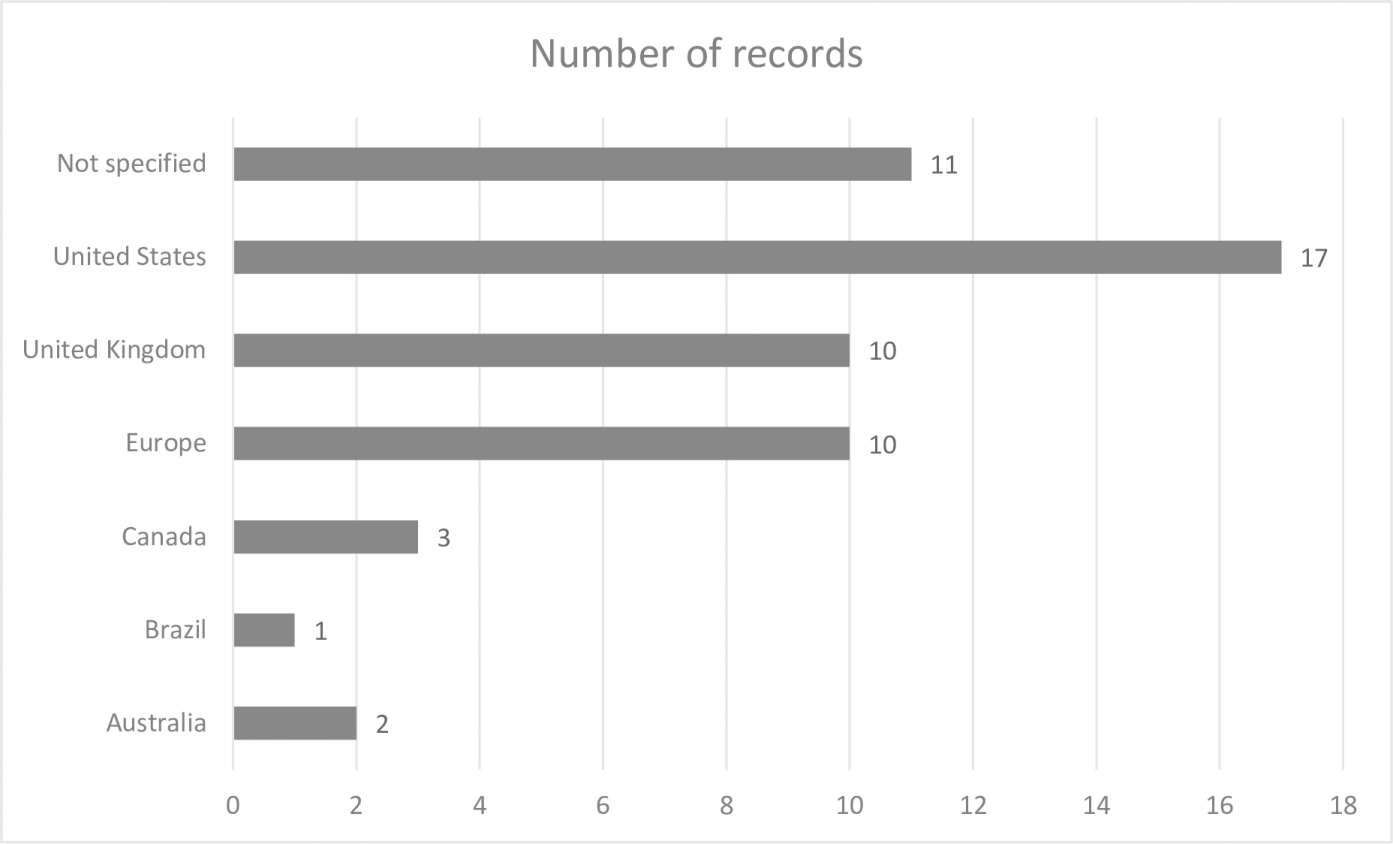
Number of records—Country wise.

**Fig 3:**
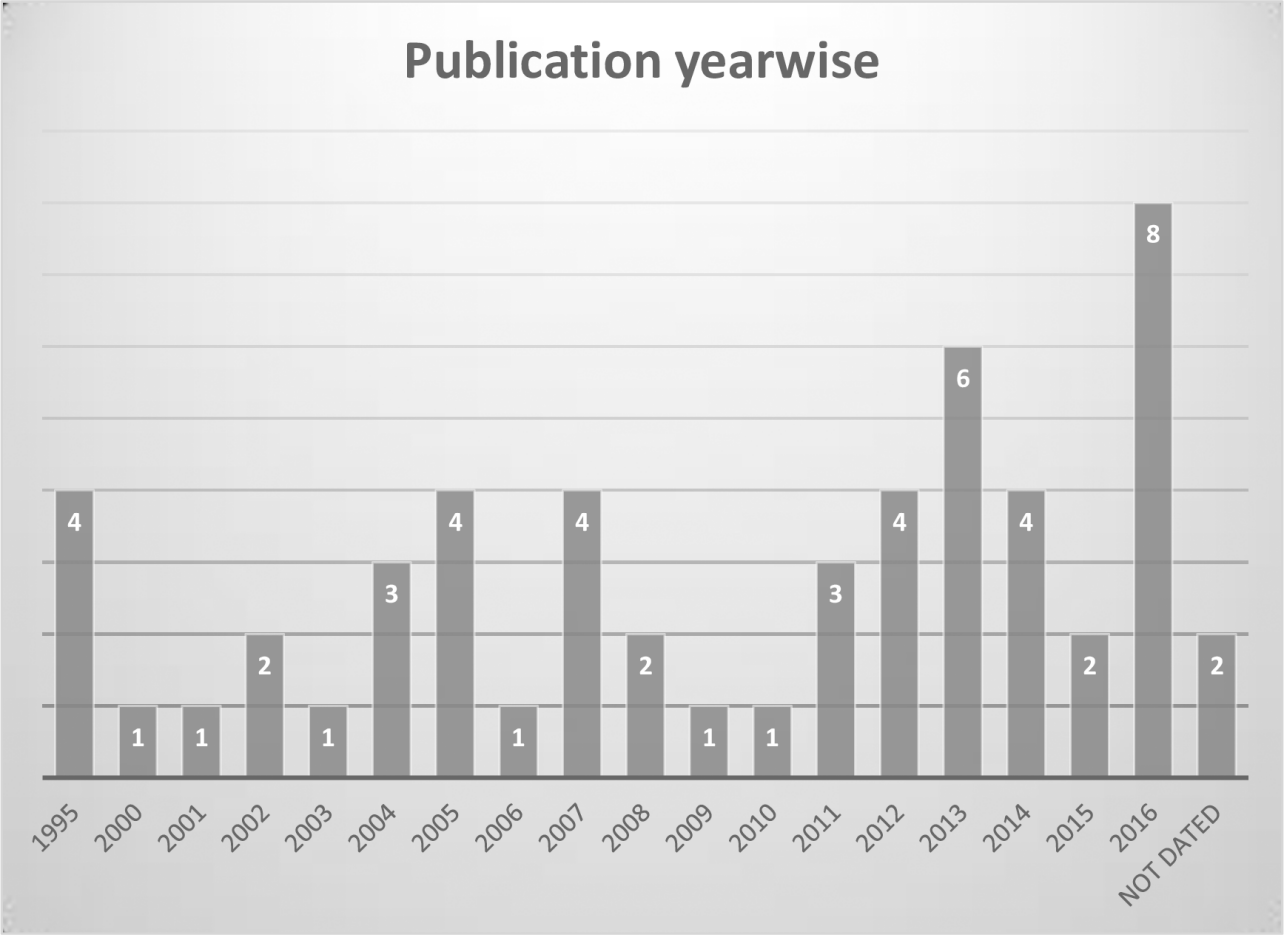
Number of sources by publication year.

Almost all of the sources were from developed nations. Most studies were based in the continent of North America (n = 20) [United States of America (n = 17) and Canada (n = 3)], and Europe (n = 20) [United Kingdom (n = 10) and other European countries (n = 10)], followed by two studies from Australia and one study from South America (Brazil) (see Fig 2). A range of 1–8 articles per year were published on topics related to experiences between 1995 and 2016 worldwide, while most records (n = 24) published in the last five years (see Fig 3).

**Fig 4:**
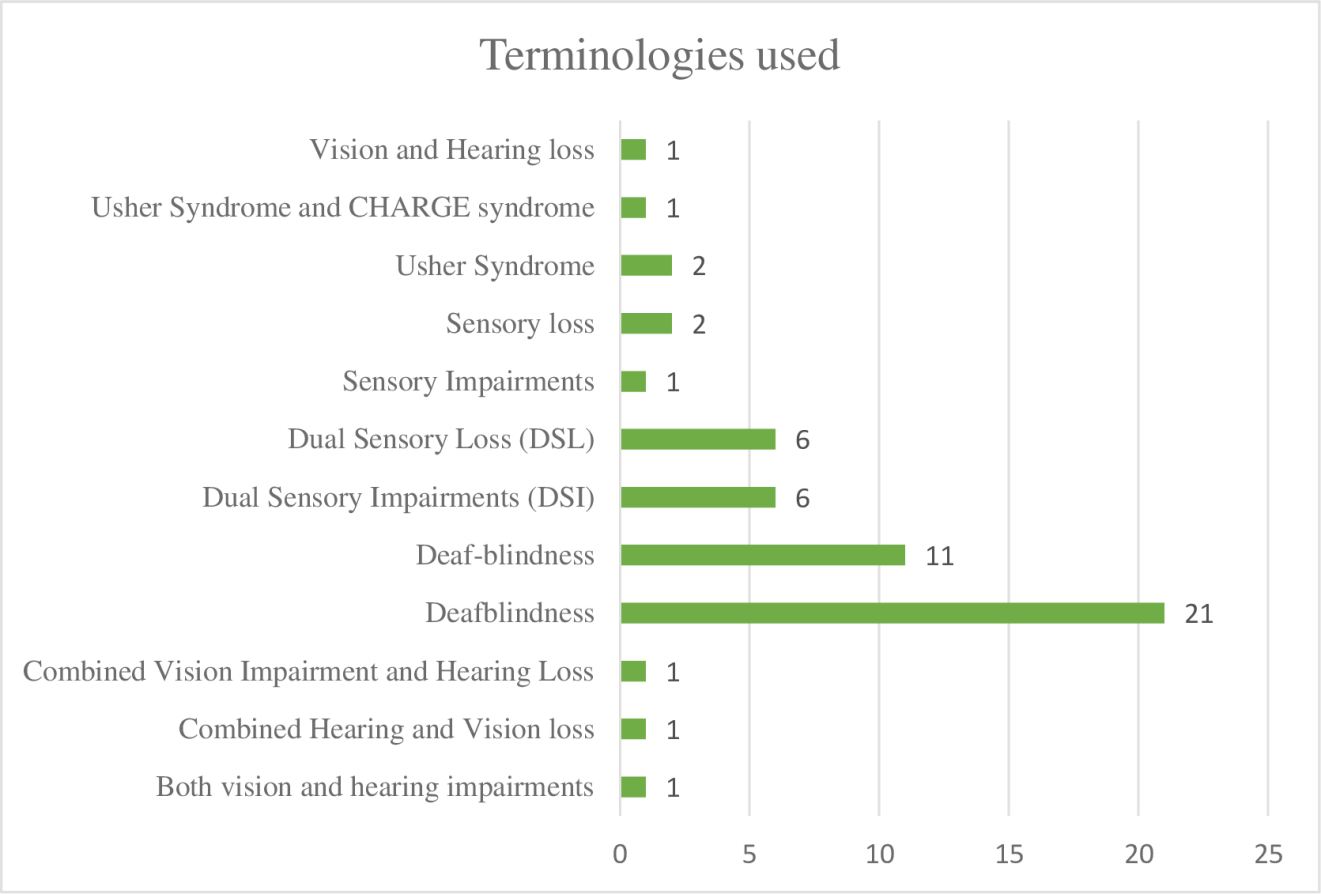
Terminologies used to describe study population.

**Fig 5:**
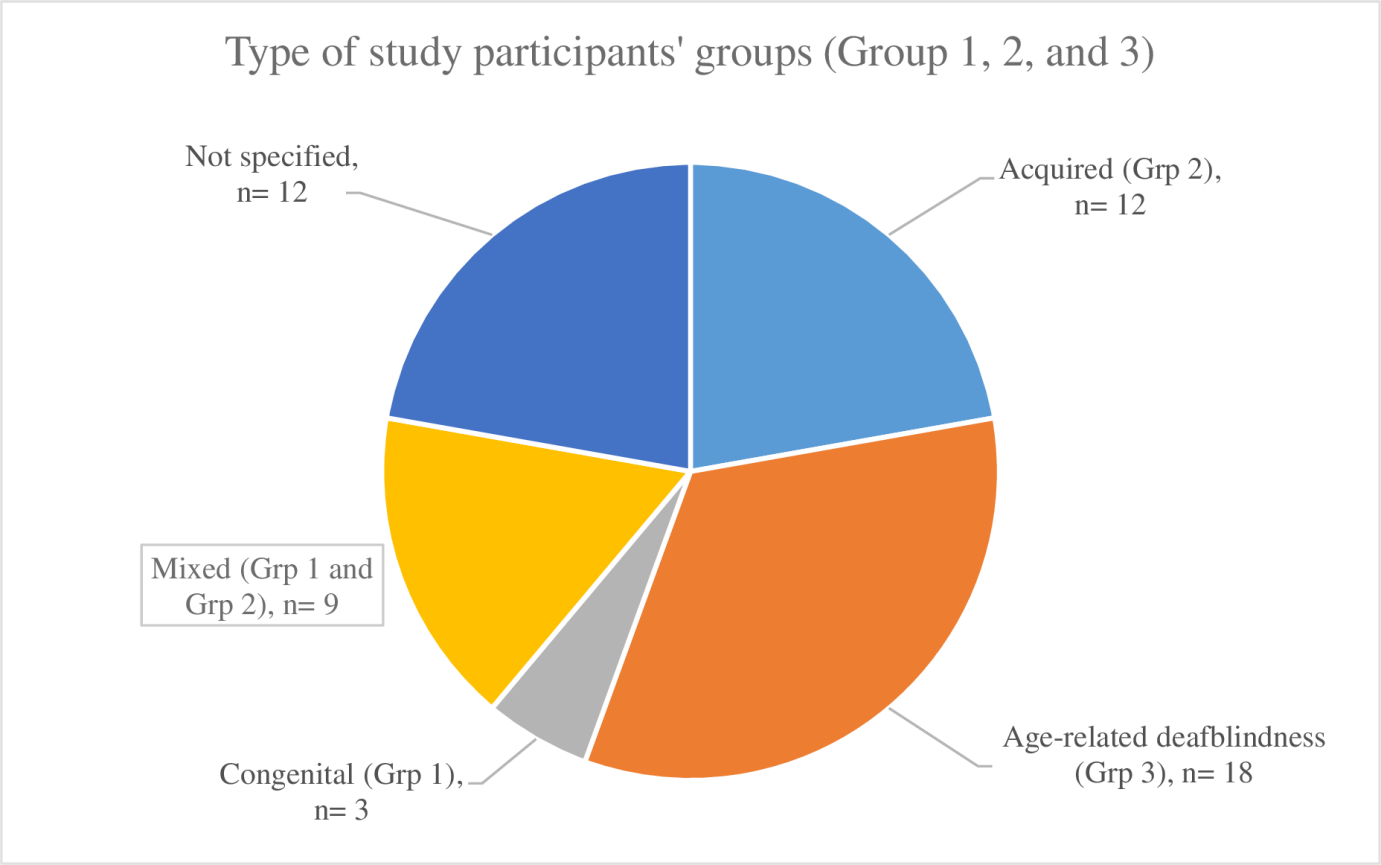
Categories of deafblindness represented across sources.

The records demonstrated variation in terminology with 11 different terms used across records to refer to the deafblind population (see Fig 4). Using the groups identified above, this scoping review found that most studies included participants from Group 3 (n = 18), followed by Group 2 (n = 12), and Group 1 (n = 3). Nine studies used mixed populations of Group 1 and Group 2 in their studies, and twelve studies did not specify the nature (congenital versus acquired) of disability of their participants (see Fig 5).

In terms of life stage, a large number of sources had participants that were adults (age between 18–65 years) (n = 23), followed by older adults (age above 65 years) (n = 13), and children (age less than 18 years) (n = 7). Most of the literature (n = 40) focused on characteristics, effects, challenges, and issues faced due to dual sensory loss or deafblindness and its impact on experiences of inclusion, social participation, engagement, physical and mental health. Some sources (n = 14) addressed experiences of dual sensory loss and incidence of depression, isolation, and suicidal tendencies in older adults (Group 3). A few sources (n = 4) also focused on experiences of leisure, recreation and physical activities.

### Major themes from the qualitative synthesis

**Fig 6:**
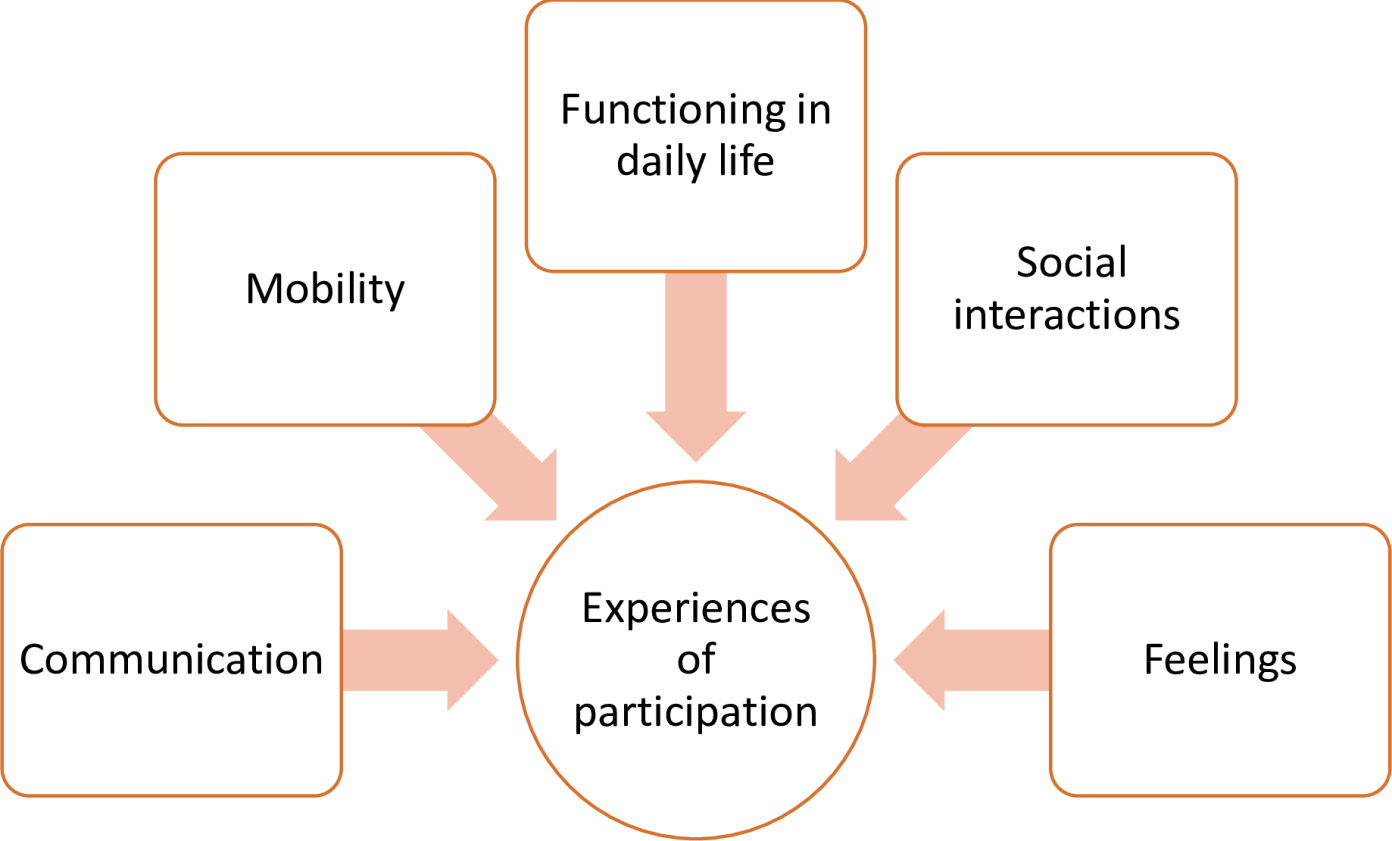
Participation of persons with deafblindness.

The themes that emerged from synthesis included experiences related to communication, mobility, functioning in daily life, social interactions, and feelings. While the Fig 6 provides a visual representation of the themes that emerged, Table 6 provides key findings across three groups. Under each of the themes discussed below, the authors first identify the experiences that are common to all three groups and then highlight only those participation experiences that were only unique to a specific group.

**Table 6:** Key findings across three groups.

Key Findings	Group 1 (congenital deafblindness)	Group 2 (acquired deafblindness)	Group 3 (age-related deafblindness)
**Communication**	experiences greater difficulty with expressive communication	experiences changing communication needs due to progressive sensory loss, reluctance to admit their hearing impairment with family, friends, and others	experiences frequent communication breakdown, inability to perceive gestural cues, embarrassment and ‘failures’
**Mobility**	Nothing specific to this group reported;	experiences embarrassment due to frequent bumping into objects/people; reported feeling stigmatized to use assistive devices such as canes in public	experiences lack of independence in indoor-outdoor mobility
**Functioning in daily life**	Nothing specific to this group reported;	experiences more difficulty in maintaining independence; tend to need more help from others to remain independent when impairment worsens; experiences reduced independence in shopping, food preparation, reading, house cleaning, watching television, reading books, listening to music, and use of technology	experiences decreased social participation with increased everyday functioning difficulties in their activities of daily living (ADL) and Instrumental ADL–dressing, meal preparation, shopping, moving around, using the phone, and managing medications
**Social interactions**	experiences limited engagement with others in society and experienced severe difficulties in social interactions than the other groups	experiences constant social isolation in life due to progressive impairments, ongoing loss of independence, and frequent worries	experiences reduced participated in social activities; avoid social contact and hence, social circle is limited.
**Feelings**	feels insecure, unsafe, and vulnerable both in and outside their homes; high risk of depression and may experience heightened vulnerability amongst deafblind groups	constantly feels worthlessness, loneliness, emptiness, uncertainty, fear of losing independence, and concerns about forming relationships, being rejected by relatives and friends, and constant concern for the future; feels depression and suicidal thoughts	feels embarrassed, offended and isolated, and have increased likelihood of depression; experiences reduced quality of life, having symptoms of anxiety or lethargy, and feeling social dissatisfaction

#### Communication

Multiple studies reported that persons with deafblindness (Group 1, 2, and 3) faced communication difficulties [3, 6, 7, 14, 29–40]. Communication breakdown was reported in two of the articles as one of the main challenges affecting social inclusion and interpersonal interactions of persons with deafblindness [36, 39]. Authors reported that persons with deafblindness felt that non-disabled people lacked knowledge about how to communicate with them, and this lack of awareness posed barriers to communication and social participation [39, 41, 42]. A woman with deafblindness in her personal narrative expressed that although there are some non-disabled people who are willing to help, they do not know how to communicate and are therefore unable to help [41].

Authors also reported that alternative means of communication such as signing, braille, print on palm, Tadoma (method of communication that involves individual with deafblindness placing their thumb on a speaker’s person's lips and their fingers along the jawline to feel their movements as they speak [6], and assistive technology such as computer/interpreter-facilitated communication means, videophone technology, and cochlear implants facilitated communication and social interactions of persons with deafblindness [32, 41, 43–45]. Additionally, strategies such as prior rehearsal of situations, use of communication repair strategies (for example asking for repetition or further clarifications from the conversational partner) and recreational activities could also be helpful in enabling successful communication [34, 46, 47]. The reviewed literature also showed that challenges in communication manifested differently for each of the three categories of deafblindness.

Group 1: Authors reported that persons with congenital deafblindness or congenital anomalies (such as CHARGE syndrome) reported greater difficulty with expressive communication than the acquired group because those with congenital deafblindness very often use signs and gestures to communicate in comparison to those with acquired where speech is prevalent mode of communication [29, 48].

Group 2: In contrast to Group 1, people with acquired deafblindness had some communication-related experiences that were unique due to their changing communication needs and progressive sensory loss, different accents, and lack of knowledge of sign language, and reluctance to admit their hearing impairment to family, friends, and others [7, 33]. One study reported that progressive loss of vision in Usher syndrome (Type 1) affects the ability to communicate with others, and causes depression and suicidal thoughts due to loneliness [49].

Group 3: A few sources focused on the distinct communication challenges and frequent communication breakdowns experienced due to age-related vision and hearing loss [34, 50]. For example, inability to perceive non-verbal and gestural cues (such as eye-gaze, facial expressions, lip-reading, and contextual cues) interferes with reception of spoken messages, and at times older adults get embarrassed by this and view themselves as ‘failures’ [34, 50]. Communication challenges for this group were primarily related to understanding and being understood, resulting in negative feelings of left out or isolation, reduced quality of life, anxiety or lethargy, and social restriction [14, 34, 35, 37, 38, 51].

#### Mobility

Challenges related to mobility and orientation were common across all three groups [29, 36, 37, 51, 52]. Key challenges included access to information from the outside world, navigating one’s environment, and using public transportation [33, 37, 41, 52, 53]. Some sources cited experiences of compromised mobility within the home and community as an impediment to participation [31, 33, 39, 41, 52].

Those with acquired condition (Group 2) often experienced embarrassment due to frequent bumping into objects/people, and rejected use of assistive devices such as canes and hearing aids in public due to perceptions of stigma [6, 32, 33, 41, 49]. With reference to older adults (Group 3), lack of independence in indoor-outdoor mobility was found to exacerbate feelings of social isolation, caused by communication challenges [37, 51, 52].

On the positive side, a few studies suggested that visual rehabilitation and training of remaining/residual senses in combination with environmental information such as the breeze of the wind, the warmth of a body, a radiator, sunlight, heavy traffic and other loud environmental sounds could improve orientation and mobility for individuals with deafblindness [32, 53].

#### Functioning in daily life

In addition to communication and mobility challenges, multiple sources reported that adults with deafblindness experienced difficulties in their daily functioning that significantly affected their social participation [14, 29, 33, 38, 52, 54–57]. Two sources identified activities of daily living such as reading, cooking, dressing, walking in the community, shopping, attending social events, getting to a doctor’s appointment, accessing information via telephone, and answering machines, as those activities where persons with deafblindness experienced challenges on regular basis [39, 54]. One survey-based study indicated high levels of unmet needs and experiences of psychological distress among men and women with deafblindness due to the loss of functional independence in their lives [58]. Another source suggested that assistive technologies and environmental adaptations could help people with dual sensory loss regain their functional independence [59]. As with the earlier two categories, articles reported variations between the three groups of persons with deafblindness.

Group 1: No themes specific to group one were identified.

Group 2: Major problems faced by this group included functional independence in shopping, food preparation, reading, house cleaning, watching television, reading books, listening to music, and use of technology [32, 33]. While considering the heterogeneity of this group, one article reported that individuals with Usher type I faced more difficulty in maintaining independence than type II; especially with progressing age (older) and worsening hearing or visual impairment, and they tend to need more help from others to be able to remain independent when impairment worsened [60].

Group 3: In the case of older adults, multiple studies reported that dual sensory loss in older adults was the strongest predictor of functional disability and need for assistance with daily activities [34, 55, 61]. A number of sources reported that older adults with dual sensory loss experienced decreased social participation with increased difficulty in performing activities of daily living (ADL) and Instrumental ADL, such as meal preparation, shopping, moving around, using the phone, and managing medications [14, 38, 52, 54–57]. In addition, this group experienced difficulty with maintaining employment, despite hearing aids. The authors attributed this particular experience to the stigma associated with sensory loss and use of hearing aids [62].

#### Social interactions and sense of isolation

Multiple articles revealed challenges with social interactions leading to loneliness and isolation [29, 33, 37, 38, 62–65]. For example, very often, people do not greet or inform the person with deafblindness of their presence and hence, persons with deafblindness have no information who is available in the same environment to interact and this lack of information restrict their social interactions. Furthermore, Simcock reported that adults with deafblindness who are ageing with congenital/acquired impairments experience high levels of isolation, due to changes in social networks and loss of friends as they get older [10]. Other sources reported that challenges in social interactions lead to social inactivity and reduced social participation in persons with deafblindness [29, 37, 38, 62, 63]. Studies also suggested approaches to facilitate social participation through group participation and social skills training; learning alternative forms of communication; using assistive technology; devising alternative leisure or recreational activities; establishing social network; and mobilizing social supports [34, 66–69].

Some of the studies reported challenges experienced by specific groups. For example:

Group 1: Specific to this group, authors reported that adults with congenital deafblindness had limited engagement with others in society and experienced severe difficulties in social interactions than the other groups [29].

Group 2: Studies reported that those with acquired deafblindness are more likely to experience constant social isolation in their lives due to the progressive impairments, ongoing loss of independence, and frequent worries (due to uncertainty about the process of the progressive loss and how to cope with it) [29, 32, 33, 67–68].

Group 3: Unique to this group, multiple studies reported that dual sensory loss very often impacted the social interactions of older adults with family and friends and as compared to the past, they participated less in social activities such as getting together with friends, shopping, going to a restaurant, and attending church and movies [35, 52, 62, 70]. Since the onset of loss, both the type and frequency of social interactions changed and they had limited their social circle by avoiding social contact [34].

#### Feelings

Multiple studies reported that adults with deafblindness experienced a myriad of feelings, including loss of independence, feelings of sadness, anger, depression, frustration, insecurity, and uncertainty about future [31, 33, 39, 71]. Authors in two survey-based studies revealed that their participants experienced psychological distress due to impairment, and developed symptoms of anxiety, stress, depression, withdrawal, and suicidal behaviors [58, 71].

Specific to the groups, in Group 1, authors reported that persons with congenital deafblindness were at high risk of depression and may experience heightened vulnerability to abuse and neglect amongst deafblind groups (group 2 and 3) [29, 72]. People, in particular women, with congenital deafblindness often feel insecure and unsafe, and described their lived experiences of feeling vulnerable both in and outside their homes, even in the context of receiving care and support [10, 72].

Group 2: With respect to this group, authors reported that persons with acquired deafblindness were more likely to experience lost roles, and faced issues in adjusting to their impairment [29]. The findings indicate that in contrast to the congenital group, "although the acquired group has the capacity to function more independently in the community, given their functional and social skills, they are more socially isolated and more likely to report feeling lonely" [29]. Authors in multiple studies found that those with Usher syndrome had constant feelings of worthlessness, loneliness, emptiness, uncertainty, fear of losing their independence, and concerns about forming relationships, being rejected by relatives and friends, and constant concern for the future [31, 33, 49, 67, 71, 73]. This particular experience was explained with “awareness of an initially relatively intact function (i.e. vision in this case)–which is progressively deteriorating–is more traumatic, compared to the case when the individuals were born without a particular function” [53].

Group 3: In contrast to the feelings experienced by Group 1 and 2, authors reported that older adults with dual sensory loss feel stigmatized due to the sensory loss and required use of assistive aids/devices [62]. Multiple studies revealed that older adults feel embarrassed, offended and isolated due to impediments to communication and social participation, and have increased likelihood of depression that leads to reduced quality of life [38, 51, 52, 56]. They found the biggest challenge as acceptance of the vision and hearing loss, and felt that “once acceptance had been achieved, the enjoyment of life could begin” [34].

## Discussion

This scoping review identified global literature on participation experiences of people with deafblindness, examining 54 articles published over the last 27 years (1990–2017). The content of these studies indicated that people with deafblindness experience significant challenges in communication, mobility, daily living functioning, and social interactions. Often, articles reported participation experiences that were common to three groups of persons with deafblindness (Group 1, 2, and 3) and some experiences that were distinct to any one group only.

### Focus of study and research methods employed

A majority of studies focused on characteristics, effects, challenges, and issues faced due to dual sensory loss or deafblindness and its impact on their experiences of inclusion, social participation, engagement, and physical and mental health. In terms of methodology employed, the majority of studies were quantitative in nature (n = 19) with use of survey data. Among those, only two studies were reported as longitudinal and three as cross-sectional study in the text. Research in this area is dominated by quantitative studies that often rely on information from proxies (family members or professionals working with people with deafblindness). Researchers have argued that there is a need for more qualitative research to develop an in-depth understanding of the needs and experiences of this population [3, 5, 7, 10, 11, 63].

### Geographic distribution and under-representation

It is clear that deafblindness research has gained interest only in recent years. Still, there is very limited literature on deafblind persons’ experiences worldwide–particularly from low and middle-income countries (LMICs). All of the 54 publications in this review were from developed nations. Furthermore, it is worth noting that the majority of research participants included in the studies were those with acquired or dual sensory loss condition (Group 2 and 3). Those with congenital deafblindness (Group 1) were rarely included as participants, as also illustrated in other studies [11]. One of the studies reported the inability to include congenital population in the study sample as their limitation due to communication difficulties in conducting interviews [33]. Consequently, individuals with congenital deafblindness are not getting opportunities to share their unique experiences and challenges.

Experiences of deafblindness in developing nations are under-represented in the literature due to one of three reasons: (a) a majority of the deafblind population in LMICs are from Group 1 as more than 100,000 children continue to be born with Congenital Rubella Syndrome each year worldwide [74, 75], (b) less deafblind-specific interventions exists in these countries [76], or (c) fewer research publications stems out from the programs that do exist, in part, due to lack of funding and/or local expertise [8, 77]. The gap warrants research publications in these contexts to inform disability policy, rehabilitation practice and research.

Another noteworthy observation is that out of 54 studies, the authors only found two personal narratives of women with deafblindness related to their life experiences [41, 43] and one article on the experiences of vulnerability by women with deafblindness [72]. The case study on the death of a young women ‘Beverley Lewis’ throws light on the fact that this population can experience heightened vulnerability to abuse and more studies are warranted to understand the complexity of vulnerability and awareness of the risk of abuse, their likelihood and severity, particularly among women with deafblindness [10, 72].

### Heterogeneity of terminologies used

Researchers in twelve of the included studies did not specify the nature (congenital versus acquired) of disability of their participants in the study. The authors also found varied definitions of deafblindness across identified studies, and eleven different terminologies were used to refer and report deafblind population in the literature. Heterogeneity of this population, variation in terminologies used, and lack of clarity in study populations (congenital/acquired) presented challenges in the generalization of the findings. The variation in terminology might distort the understanding of this condition among researchers and professionals. For instance, it can lead to an assumption of deafblindness as a rare condition and hinder identification of people having combined hearing and vision impairment in society.

Available literature lacks a clear definition of dual sensory loss or deafblindness [1, 3, 11] and that might be a reason behind this lack of clarity among researchers while reporting their study sample characteristics. Similar findings were reported by Wittich and colleagues [2] in their study on existing terminology and its use related to combined vision and hearing loss in both the research community and among professionals working in the field of rehabilitation. They emphasized the need to harmonize terminology across practitioners and researchers on deafblindness to enable clarity in communication between different stakeholders and facilitate knowledge translation of research findings in their respective fields [11].

### Heterogeneity of the population and participation experiences

The heterogeneity of this condition leads to varied experiences related to participation and manifests in the varied forms of communication difficulties, mobility restrictions, decline in functioning, social isolation, and the myriad of feelings [6, 7, 58]. Communication, which plays a key role in social participation for any individual, emerged as one of the most significant domains affected by deafblindness and is more likely to cause difficulties in social interactions [78].

In general, the available scientific literature is silent on the experiences of people with congenital deafblindness. The authors found very few studies that clearly discussed the experiences of persons with congenital deafblindness in relation to their involvement in daily life. [29, 72, 79]. Although the study by Dalby and colleagues [29] revealed that people with congenital deafblindness experienced more challenges in communication, mobility, activities of daily living, and social interactions with others than did the acquired group, it is also important to understand how they are more vulnerable to abuse and neglect [29, 72].

In case of Group 2 (those with acquired impairment), the struggles were more to adjust to progressing visual or hearing loss and its impact on their independence in daily life. Those with acquired deafblindness are more likely to experience lost roles and face issues in adjusting to their acquired dual sensory impairment and develop feelings of loneliness. Even within Group 2, people who have progressive vision loss versus progressive hearing loss had distinct experiences of challenges of participation. For example, those with progressive vision loss face challenges in adapting to the changing communication needs, ongoing loss of independence, and increased dependence on others to assist in mobility and navigating the environment [33, 41, 49, 67]. Whereas those with progressive hearing loss reported their fear of not knowing about an emergency situation, significant difficulties in communication due to loss of audible inputs and required change to learn to use hearing aids or cochlear implants [60]. The feeling of constant insecurity and lack of belongingness (being included and being accepted) plays a critical role in shaping their experiences of participation in the world [80].

In the case of Group 3, the older adults with dual sensory loss experience distinct problems of adjusting to sensory loss, frustration, depression, anxiety, lethargy and social dissatisfaction [34, 37]. They are more at risk of mental health concerns as compared to other two groups and do feel stigmatized in the use of assistive aids for hearing and mobility [6]. They also experienced two folds struggles–first due to communication breakdowns caused by co-existence of age-related vision and hearing loss, and second due to difficulty in learning to use specific technology (for example, computer, iPad and cellular telephone) in later stages of life which could overcome their communication challenges [34, 37].

Overall, this scoping study synthesizes the literature on participation experiences of persons with deafblindness and summarizes a range of evidence that could inform the programs and policies for rehabilitation of persons with deafblindness. The study documents that persons with deafblindness, regardless of their group (congenital, acquired or age-related), experience difficulty in communication, mobility, functioning, and access to information; and feel socially isolated, insecure and uncertain about their future. They are also at high risk of developing mental health issues as their age advances, and that may further worsen their condition and restricts their social participation in society. Therefore, rehabilitation interventions should be designed keeping in the mind these specific and unique challenges faced by persons with deafblindness.

### Limitations

One major limitation for this scoping study is related to generalizability of its findings for the deafblind population from developing nations. Given the geographical location of the included studies are from developed nations, it is likely that a bias exists, reflecting the experiences of persons with deafblindness from developed nations only, especially from North America and Europe. However, given that the authors were unable to find peer-reviewed and non-peer reviewed publications from LMIC on the participation experiences of persons with deafblindness, this study might be a helpful first step in helping LMIC stakeholders understand the experiences of people with deafblindness. Future research should explore their experiences in the LMIC contexts.

This study only reported results from articles published in English, which may have contributed to the absence of LMIC publications, as those articles may indeed exist in the local context and local language. Conducting the literature search in languages other than English would permit more confident claims regarding the comprehensiveness of the findings in this scoping review. Additionally, while this review was focused on experiences of persons with deafblindness, it is possible that including the perspectives of caregivers and professionals on the same focus might have enriched and complemented the findings [12]. Also this was not a systematic review so methodological rigor of studies was not evaluated.

## Conclusions

The United Nations 2030 Agenda for the Sustainable Development Goals reiterates the principle of “leaving no one behind”, and the United Nations Convention on the Rights of Persons with Disabilities (2006) promotes the goal of maximizing participation of persons with disabilities, including deafblindness, in society. However, individuals with deafblindness are often absent in rehabilitation research due to the complex nature of the disability and methodological challenges involved with recruitment and data collection. Therefore, conducting research on deafblindness and raising awareness of this distinct disability is imperative so that persons with deafblindness receive needed deafblind-specific rehabilitation services to enhance their participation and quality of life.

This review demonstrated that persons with deafblindness, regardless of the nature of their impairment, experience significant challenges in participation in day-to-day lives (especially in communication, mobility and social interactions) and are at high risk of developing mental health issues as their age advances. Researchers very often associated the challenging participation experiences with the impairment rather than the environmental factors, locating problem within the individual. Participation experiences of persons with deafblindness are shaped by dynamic interactions between personal factors (such as onset and type of impairments) and environmental influences (such as attitude, technology, and supports). A better understanding of these participation experiences may help rehabilitation professionals in placing emphasis on affected participation domains to design services that strive to enhance participation of persons with deafblindness. Moreover, the review of global deafblind-specific literature reveals that there is an absence of research literature on deafblindness from the LMIC and warrants research in these contexts to inform policy, practice and research.

## Supporting information

S1 File PRISMA 2009 checklist. (DOC)
